# Telehealth for Children With Epilepsy Is Effective and Reduces Anxiety Independent of Healthcare Setting

**DOI:** 10.3389/fped.2021.642381

**Published:** 2021-06-10

**Authors:** Kerstin Alexandra Klotz, Felippe Borlot, Morris H. Scantlebury, Eric T. Payne, Juan Pablo Appendino, Jan Schönberger, Julia Jacobs

**Affiliations:** ^1^Department of Neuropediatrics and Muscle Disorders, Faculty of Medicine, Medical Center-University of Freiburg, University of Freiburg, Freiburg, Germany; ^2^Faculty of Medicine, Epilepsy Center, Medical Center-University of Freiburg, University of Freiburg, Freiburg, Germany; ^3^Berta-Ottenstein-Programme, Faculty of Medicine, University of Freiburg, Freiburg, Germany; ^4^Cumming School of Medicine, Alberta Children's Hospital Research Institute and Hotchkiss Brain Institute, University of Calgary, Calgary, AB, Canada; ^5^Section of Neurology, Department of Pediatrics, Alberta Children's Hospital, Cumming School of Medicine, University of Calgary, Calgary, AB, Canada

**Keywords:** epilepsy, telehealth, anxiety, COVID pandemic, seizures, health system

## Abstract

**Objectives:** The use of telemedicine has grown exponentially as an alternative to providing care to patients with epilepsy during the pandemic. We investigated the impact of the current pandemic among children with epilepsy from two distinct pediatric epilepsy centers. We also compared perceptions among those who received telemedicine against those who did not.

**Methods:** We developed a questionnaire and invited families followed in Freiburg, Germany, and Calgary, Alberta, Canada, to participate during the initial 9 months of the pandemic. The survey contained 32 questions, 10 of which were stratified according to telemedicine exposure.

**Results:** One hundred twenty-six families (80 in Freiburg, 46 in Calgary) participated, and 40.3% received telemedicine care. Most children (mean age 10.4 years, *SD* 5.1) had chronic epilepsy but poorly controlled seizures. Negative impacts were reported by 36 and 65% of families who had to reschedule appointments for visits and diagnostics, respectively. Nearly two-thirds of families reported no change in seizure frequency, while 18.2% reported either worsening or improvement of seizures. Although most families did not note behavioral changes, 28.2% reported behavior worsening. Families who received telemedicine care had a statistically significant reduction of parental self-reported anxiety level after virtual visits compared to those who did not experience telemedicine. Families with telemedicine consultations were more likely to consider future virtual care (84 vs. 65.2% of those without), even after the pandemic. Patient data safety, easy access to specialized services, and consistency with the same healthcare provider were graded as important in both centers, while a shorter waiting time was most relevant in Calgary.

**Conclusion:** In our cohort, some children with epilepsy experienced increased seizures and worsening behavior during the first 9 months of the current pandemic. In addition, our data suggest that telemedicine might reduce parental anxiety symptoms, and families who experienced telehealth were more positive and open to similar appointments in the future.

## Introduction

The coronavirus disease 2019 (COVID-19) pandemic has had a substantial impact on the way healthcare providers and institutions deliver care worldwide. Face-to-face outpatient services were abruptly closed, and many families of chronically ill children were left without the expected support. Indeed, among children with epilepsy, regular medical support is of paramount importance given the unpredictability of seizures and the complex care many of these patients require, including developmental and behavioral challenges (1).

Telemedicine use has grown exponentially as an option for epilepsy care and decreases the risk of COVID-19 exposure for families and healthcare providers. Even though telemedicine has been successfully used to provide epilepsy care for over a decade in some centers (2, 3), it was underutilized in epilepsy care before the pandemic (4). Initially designed to provide care in rural and remote areas, telemedicine effectiveness and patients' and providers' high satisfaction rates have encouraged its implementation in several centers (3, 5–7). In addition, virtual visits may also save costs for patients (3). Furthermore, with communication tools becoming easily accessible throughout the world, many patients and providers have expressed their willingness to incorporate both in-person and virtual appointments (7, 8).

Although recent studies have shown that telemedicine is feasible and effective in child neurology and epilepsy care (3, 5, 9), scattered data are available for the current pandemic, including how parents of children with epilepsy perceive the pandemic is impacting their child's overall health, seizures, and behavior and parental anxiety levels and whether direct exposure to telemedicine impacts these perceptions.

The objectives of this study were (i) to investigate the consequences of the pandemic as reported by families followed in two pediatric epilepsy centers, one in Canada and one in Germany; (ii) to compare families with and without telemedicine experience during the first 9 months of the pandemic; and, finally; (iii) to learn what families consider important when it comes to telemedicine.

## Materials and Methods

We developed a structured and stratified questionnaire using an online survey tool and invited families with outpatient appointments scheduled between February and October 2020 to participate. Our questionnaire was developed to obtain an overview of parents' perception in the way we had to adapt delivery of care during the pandemic and their feelings about the pandemic-related acute measures, rather than assessing specific intervention effects of telemedicine. For participating, patients must have been diagnosed with epilepsy by a pediatric neurologist, according to the International League Against Epilepsy (ILAE) criteria (10). A short introduction explained the term “telehealth” to all participants. The survey contained 32 questions with the last 10 modified depending on whether the patient had or had not participated in a telemedicine consultation. The survey started with questions about the patient's age and epilepsy history, including age at first seizure, current treatment, seizure frequency, and routine epilepsy-care schedule. The second part included questions about scheduled appointments and diagnostics during the pandemic, the impact of changed or canceled appointments on the child's health, and the impact of pandemic-related restrictions on the child's health. The third part focused on telemedicine, including what technical equipment was available in their household. Depending on whether the patients had telemedicine care during the pandemic, the questions were stratified. From those participants reporting previous telemedicine appointments, information about the following topics were asked: provider specialty using telemedicine, type of medium used, and whether the appointment was considered helpful regarding several aspects of the child's epilepsy. For families without previous telemedicine appointments during the pandemic, the survey included questions about their media preferences, expectations, and whom they would prefer to conduct a virtual health consultation. Finally, all participants were asked about the importance of data protection, accessibility, other aspects of telehealth consultations, preferred media, and reasons to consider telehealth in the future even after the pandemic. This survey was approved by the ethics committees from both Albert-Ludwigs-Universität Freiburg (No. 68/18) in Germany and Alberta Children's Hospital Research Institute (REB20-0670) in Canada. The English and German versions of the survey are available in the Supplementary Material (Survey Telehealth English version and Survey Telehealth German version).

Descriptive statistical analysis was performed with GraphPad Prism (V. 9.0, GraphPad Software, San Diego, CA, USA). Categorical variables are presented in absolute numbers and percentages and quantitative data as means and standard deviations. Percentages apply to the number of answers for any given question. A Fisher exact test was used for group comparisons of categorical and ordinal values and a Mann–Whitney U test for comparison of numerical values. *P*-values ≤ 0.05 were regarded as statistically significant.

## Results

### Demographics, Cancelations, and Impact of the Pandemic in the Study Population

Overall, 80 families in Freiburg and 46 families in Calgary answered the questionnaire. The response rate was 41.5% (126/303). Of those, 119 questionnaires were complete and could be included for analysis. For details about patients' epilepsy and routine epilepsy care, see Table 1. Most participants reported more than one available technical equipment for telemedicine in their household, and the following devices were available: phone 94.6% (*n* = 106), Wi-Fi 93.8% (*n* = 105), tablet with camera 86.8% (*n* = 97), personal computer with camera 83.0% (*n* = 93), and chat programs 80.4% (*n* = 90).

**Table 1:** Patient characteristics.

	**All**	**Freiburg**	**Calgary**	
	**(*n* = 126)**	***(n* = 80)**	**(*n* = 46)**	***P*-value**
**Age in years mean (*****SD*****)**	10.4 (5.1)	10.0 (5.2)	11.2 (4.8)	0.08
**First seizure** ***n*** **(%)**				
Within 1 month	14 (11.3)	10 (12.8)	4 (8.7)	0.57
Within 1 year	9 (7.3)	2 (2.6)	7 (15.2)	0.01
Within 1–5 years	40 (32.3)	31 (39.7)	9 (19.6)	0.03
>5 Years ago	61 (49.1)	35 (44.9)	26 (56.5)	0.27
**Seizures within 12 months**	93 (74.4)	57 (73.1)	36 (76.6)	0.53
**Currently on any ASM**	118 (95.2)	75 (96.2)	43 (93.5)	1.0
**Change of therapy within the last 12 months**	67 (54.0)	41 (52.6)	26 (56.2)	0.58
**Seizure frequency**				
Daily	33 (37.1)	20 (37.0)	13 (37.2)	0.26
Weekly	14 (15.7)	8 (14.8)	6 (17.1)	0.36
Monthly	22 (24.7)	15 (27.8)	7 (20.0)	1.0
Less than monthly	20 (22.5)	11 (20.4)	9 (25.7)	0.18
**Tonic–clonic seizures** ***n*** **(%)**				
Never	39 (32.2)	25 (32.9)	14 (31.1)	1.0
Past only	47 (38.8)	29 (38.2)	18 (40.0)	0.57
Recently	35 (28.9)	22 (28.9)	13 (28.9)	1.0
**History of prolonged seizures** ***n*** **(%)**	49 (40.2)	36 (47.4)	13 (28.3)	0.09
**Epilepsy care**				
By pediatric neurologist	114 (94.2)	71 (93.4)	43 (95.6)	1.0
By pediatrician	6 (5.0)	4 (5.3)	2 (4.4)	1.0
By family physician	1 (0.8)	1 (1.3)	0	1.0
**Scheduled outpatient appointments**				
Monthly	6 (5.0)	6 (8.0)	0	0.08
3–4 Times per year	48 (39.7)	30 (40.0)	18 (39.1)	1.0
Twice per year	49 (40.4)	33 (44.0)	16 (34.8)	0.35
Annually or less	18 (14.9)	6 (8.0)	12 (26.1)	0.009

*Percentages calculated by number of responses for each question. ASM, anti-seizure medication*.

At the onset of the pandemic, 63.8% (*n* = 76/119) had an outpatient appointment scheduled or were waiting for an appointment. In 32.9% (*n* = 25/76) of those, the appointment could take place as scheduled, in 40.8%, it was canceled and replaced by a virtual consultation; in 14.5%, it was canceled and replaced by a later appointment; and in 11.8%, it was canceled without an alternative appointment. For those 51 cases that a scheduled appointment had to be changed, the majority (62%) felt that it did not impact their child's health, but 36% felt a negative impact either because treatment or planning of further diagnostics was delayed (28%) or because important questions were not addressed (8%). Diagnostic tests were scheduled in 26.9% of patients (*n* = 32/119) at the beginning of the pandemic, including electroencephalogram (EEG; *n* = 11), video-EEG monitoring (*n* = 8), MRI (*n* = 3), PET (*n* = 1), and others (renal ultrasound, *n* = 1; sleep study, *n* = 1; ophthalmologist appointment, *n* = 1). Of those, diagnostic tests took place as scheduled in 37.5%, but in 43.8%, these investigations were canceled with a postponed appointment; in 18.8%, canceled tests were left without an alternative. In most cases (65%), parents were concerned about a negative impact on their child's health if diagnostic appointments were canceled or postponed mainly because diagnostics were necessary to change or initiate a certain treatment (69%). During the pandemic-related restrictions, the majority of parents observed no change in overall health (59.5%, *n* = 47/79), seizure frequency (63.6%, *n* = 49/77), or behavior (52.6%, *n* = 41/78); whereas 25.3% (*n* = 20/79) observed an improvement in overall health, 18.2% (*n* = 14/77) in seizure frequency, and 19.2% (*n* = 15/78) in behavior. Worsening of overall health was reported in 15.2% (*n* = 12/79), of seizure frequency in 18.2% (*n* = 14/77), and of behavior in 28.2% (*n* = 22/79). Nearly one-third of parents reported anxiety that their child's epilepsy would worsen during the pandemic (30.7%, 35/114).

### Telemedicine Experience Vs. No Telemedicine Experience

Overall, 40.3% (*n* = 48/119) of participants received telemedicine care, and some of these patients were seen more than once and used different media. Appointments regarding the epilepsy were mainly with pediatric neurologists (76.6%, *n* = 36), and in some cases, with their pediatrician (23.4%), family physician (12.8%), a registered nurse (12.8%), or other subspecialties including neurosurgeons or metabolic clinics (19.1%). Phone was the medium used in most telehealth appointments (83%, *n* = 39), whereas Internet services (specialized telemedicine platform, email, etc.) were used by 57.4%. Almost all families perceived the virtual consultation as helpful (95.5%, *n* = 42/44). Within the comments, parents stated that it was helpful because “*many aspects, e.g., adapting the medication or counseling can be easily done over the phone*.” Others stated that it was not helpful because “*telehealth is less personal and it is hard to build a relationship, especially for autistic or severely impaired children*.” Compared to in-person appointments, 47.7% (*n* = 21/44) found telemedicine efficient, 38.6% (*n* = 17) nearly as efficient, and 13.6% (*n* = 6) found it only partially helpful. Thirty-two percent (*n* = 14/44) had to come to the hospital despite virtual visits because (i) it was recommended during the telemedicine consultation (42.8%), (ii) acute deterioration (28.6%), or (iii) unrelated to epilepsy (28.6%). For further telemedicine consultations, most families would prefer phone calls (46.5%, *n* = 20) or online video chats (41.9%); only 7.0% would prefer a telehealth platform, and none would prefer the consultation to be *via* email.

At the time of the survey, 59.6% (*n* = 71/119) of all families had not experienced telemedicine. If this modality was needed and available in the near future, most families expected it to be with a pediatric neurologist (89.8%, *n* = 62) and *via* Internet services (58.5%) or *via* phone (48.6%). Sixty-seven percent expected it to be helpful, and reasons stated were that “*telehealth is an option during quarantine and lockdown to get in contact with a medical professional*.” Of those expecting telehealth not to be helpful, comments included “*no diagnostics possible*” and the fact that “*contact would be mainly between parents and physician, not so much with the child*.” Virtual appointments were expected to be as efficient in 17.1% (*n* = 12/70), nearly as efficient in 38.6%, partially helpful in 35.7%, and not helpful in 8.6%.

### Parental Self-Reported Anxiety Levels, Differences Between the Two Centers, and Other Relevant Aspects of Telemedicine

**Figure 1:**
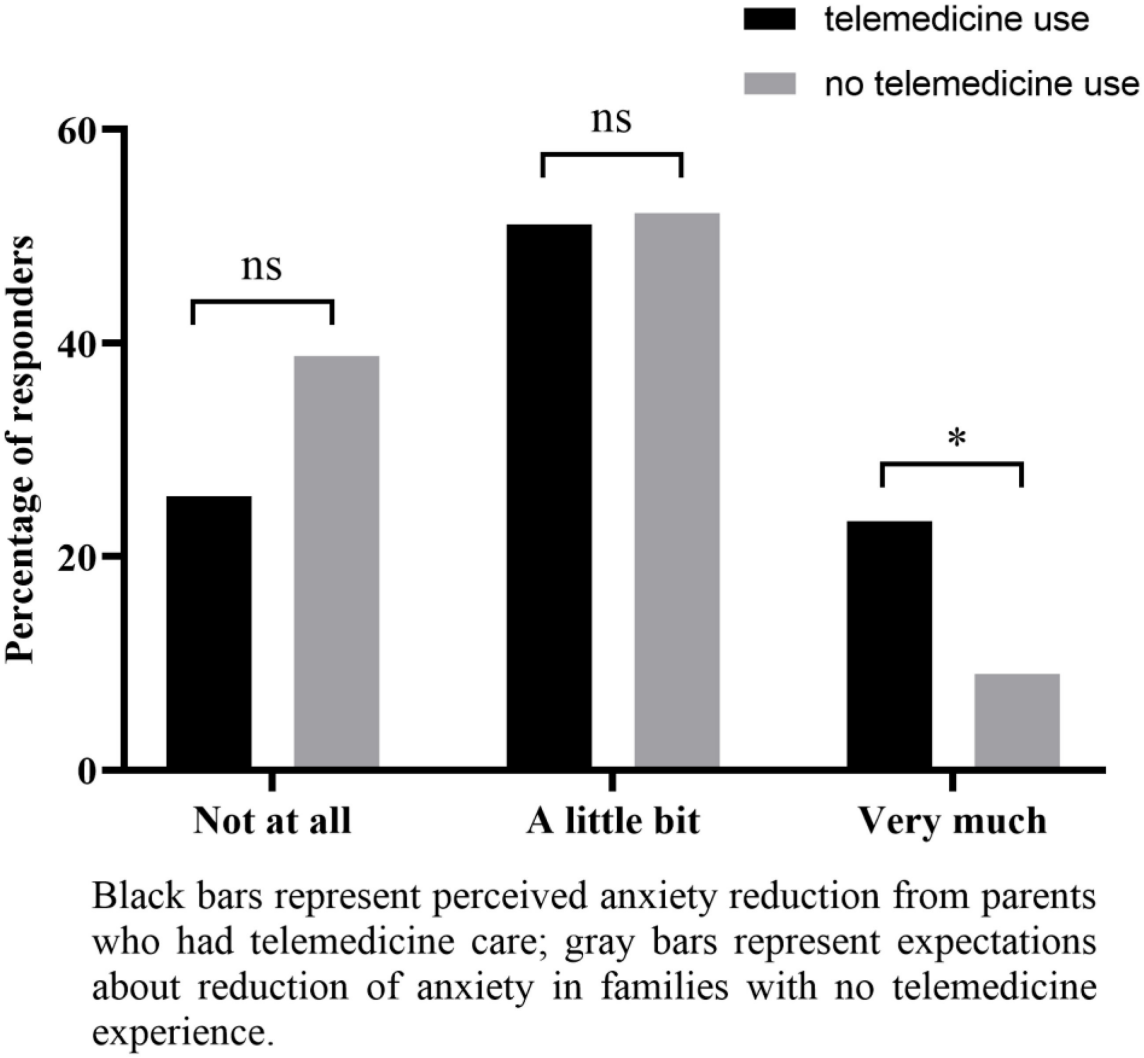
Perceived reduction of anxiety because of telehealth consultation (parents who received telemedicine care) and expected reduction of anxiety in case of a future telehealth consultation (parents with no telemedicine experience). *P-value ≤ 0.05; ns, not significant.

Related to parental anxiety levels, relevant differences were seen between families with and without previous telemedicine experience. In both groups, roughly 30% expected that anxiety symptoms could worsen during the pandemic. However, parents with telemedicine experience reported a reduction of anxiety after a telemedicine consultation, while the great majority of parents with no experience would not believe that their anxiety levels would reduce very much after a virtual consultation (Figure 1). Also, 84% of families with previous telemedicine consultations (vs. 65.2% of those with no telemedicine experience) would be more likely to consider changing all or some of their appointments to telemedicine in the future, even after the pandemic. Conversely, 34.8% of families who did not experience virtual appointments (vs. only 15.9% of those who did, *P* = 0.03) would not consider changing all or most of their future appointments (Table 2).

**Table 2:** Consideration of future telemedicine consultations.

**Would you consider changing all or most of your appointments to this form if possible, even after the pandemic?**			
	**Previous use of telemedicine (*****n*** **=** **44)**	**No previous use of telemedicine (*****n*** **=** **69)**	***P*****-value**
Yes, all	4 (9.1)	8 (11.6)	0.76
Yes, some	33 (75.0)	37 (53.6)	0.03
No, I prefer personal appointments	7 (15.9)	24 (34.8)	0.03

**Figure 2:**
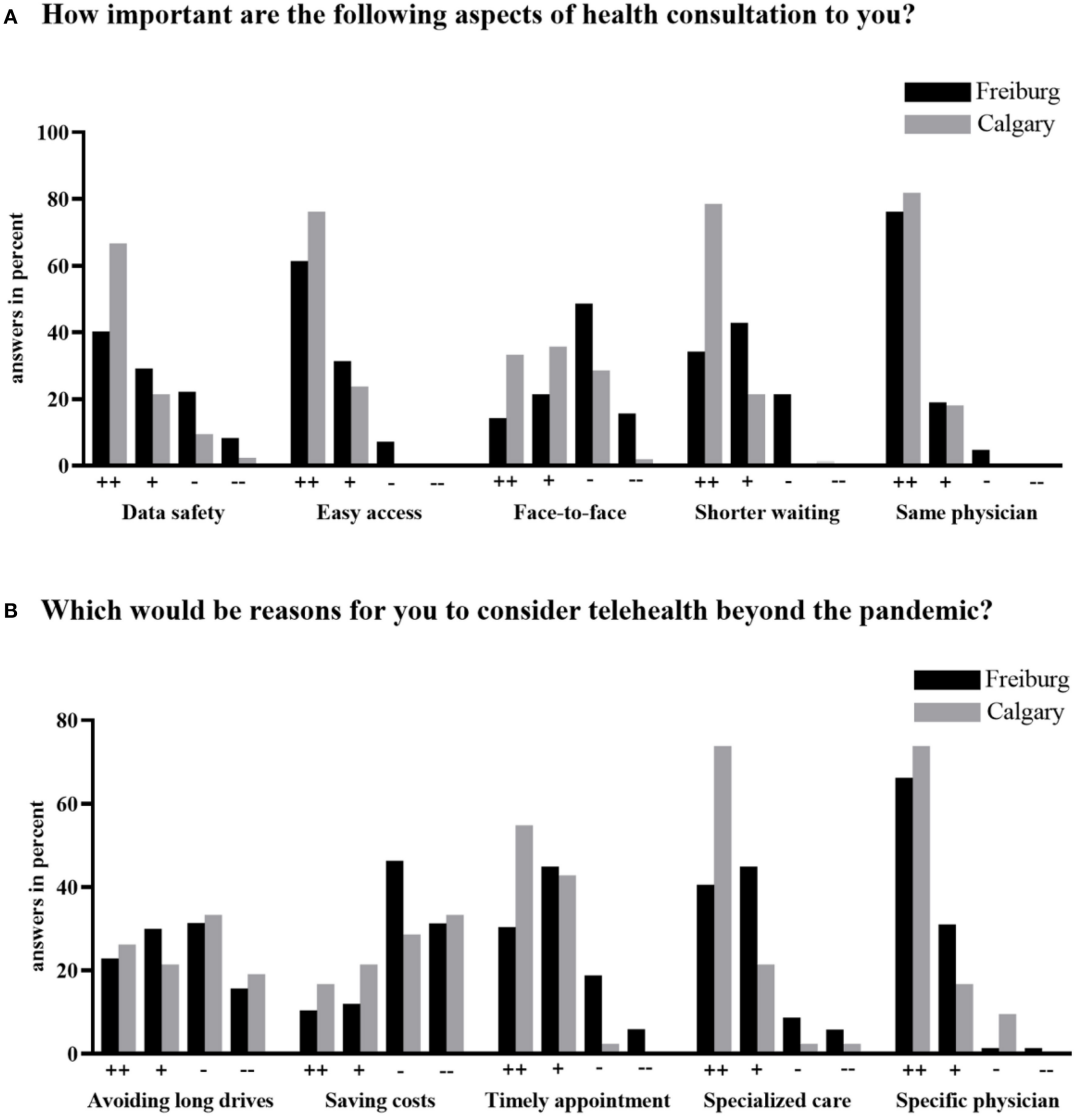
Importance of health consultations in general **(A)** and reasons to consider telehealth beyond the time of the pandemic **(B)** reported by families in Freiburg, Germany (*n* = 72) and Calgary, Alberta, Canada (*n* = 42).

There were a few differences between families from Freiburg and families from Calgary. From the patient characteristics (Table 1), there were statistically significant differences in regard to scheduled outpatient appointments with neurology. Monthly outpatient visits were reported only in Freiburg (8 vs. 0%, *P* = 0.08), while 26% of Calgary families reported annual or less frequent visits (vs. 8% in Freiburg, *P* = 0.009). When asked how long they felt they could handle their child's disease with virtual appointments only before presenting to an emergency department, 8.7% of families from Freiburg reported a time frame of <1 month, 44.9% between 2 and 6 months, 34.8% between 6 and 12 months, and 11.6% more than 12 months. In Calgary, 2.4% of families reported a time frame of <1 month, 9.5% between 2 and 6 months, 45.2% between 6 and 12 months, and 42.9% more than 12 months. There were also differences between the two centers regarding the importance of health consultations and reasons to consider telemedicine even after the pandemic (Figures 2A,B).

## Discussion

Our study gathered 126 responses from two distinct pediatric epilepsy centers during the first 9 months of the COVID-19 pandemic. Although geographically apart, the patient populations studied were similar in the two centers consisting of children (mean age 10.4 years, *SD* 5.1) with chronic epilepsy but poorly controlled seizures in the past 12 months, many of whom required recent medication changes. In keeping with another survey carried out in North America, technology access telemedicine was not a limitation to our patients (7). However, one should consider limited access to Internet as a relevant barrier for virtual clinics in underdeveloped countries, and telephone contact should be prioritized (11).

One small difference we noted between the two centers was the frequency patients are seen by their neurologists (Table 1). In Calgary, 26.1% of children are seen yearly or less frequently compared to 8% of children in Freiburg. This finding purely reflects the wait-list aspects from both centers, showing that follow-up visits are usually more frequent in Germany. One explanation for this could be that registered nurses and family practitioners are often involved in epilepsy patient care in Calgary. Also, this slight difference reflects families' perception about their ability to manage their child's disease only virtually before having to come to the emergency department. In Freiburg, most responders felt that they could handle it for 2–6 months, and only 11.6% responded more than 12 months. On the other hand, the majority of families from Calgary responded 6–12 months, closely followed by a time frame of 12 months reported by more than 40%. Considering similar patient populations, this difference might suggest that because of longer wait times to see a neurologist regularly, families may develop more autonomy and confidence over time, as well as less need to visit emergency services.

For families that had to cancel or reschedule outpatient visits, more than a third were concerned about negative impacts on their child's health. Furthermore, for those who had diagnostic tests rescheduled or canceled, nearly two-thirds felt the same way. Another study from Germany (12) has also pointed to epilepsy patient frustrations and concerns after the latest changes in the way care is currently delivered. Only a minority of patients (12.5%) followed in a tertiary epilepsy center in Frankfurt showed lack of understanding or reacted with anger after having their in-person visits canceled. As our patients stated, some of their concerns were also related to diagnostic and potential treatment delays. We believe that under normal circumstances, perhaps these abrupt visit cancelations would not impact the majority of people; however, given all the psychological distress caused by the current outbreak (13), services might consider offering coping mechanism strategies for families should future abrupt cancelations be required.

Even though one-third of parents revealed anxiety with the possibility of increased seizures during the pandemic, overall, this was not commonly noted. While worsening of behavior was reported by 28.2% of families, increased seizures and worsening of overall health were, respectively, noted in 18.2 and 15.2%. Similarly, from 109 telemedicine appointments in Frankfurt, 14.7% also reported increased seizure frequency (12). At this point, it is unclear whether these reported symptoms can be truly related to stress or any other pandemic-related cause, given the fluctuations usually seen in patients with chronic and uncontrolled epilepsies. Ideally, a baseline standardized assessment (including seizure frequency, emergency department visits, and rescue medication utilization) prior and during the pandemic would be necessary to evaluate whether an association exists between restrictive measures and seizure burden. However, consistent with our study, among 255 adult epilepsy patients studied during the first month of confinement in Spain, only 10% reported an increase in seizure frequency (14). These authors noted a higher risk of increased seizures due to tumor-related seizures, medically refractory epilepsy, insomnia, fear of epilepsy, and income reduction.

After comparing families with and without telemedicine experience, we have found statistically significant differences related to self-reported parental anxiety after experiencing virtual visits, albeit no objective scales were used. While only 9% of parents who have not tried telemedicine expected anxiety to decrease “very much,” 20% of those who had tried telemedicine declared that their anxiety levels were reduced “very much.” These data reinforce the efficacy of telemedicine in epilepsy care compared to face-to-face visits not only by epilepsy parameters such as seizure frequency, emergency room visits, and hospital admissions (2) but also addressing parental concerns.

Further analyses of data comparison from families with and without telemedicine experience have shown that while the former group would be willing to switch all or most of their future appointments to virtual visits, the latter group was opposed to this change. This reluctance observed in families without prior telemedicine experience also reflects their impression that virtual visits are only partially (35.7%) or not (8.6%) helpful, as opposed to nearly 90% of families who have experienced telemedicine and reported this modality as nearly or as efficient as face-to-face visits. The impression from our families who have been exposed to telemedicine supports several other research data. Twelve years before the current outbreak, a pilot study investigated the satisfaction with telemedicine among epilepsy patients from rural areas of Alberta (3). Aside from being very satisfied with telemedicine, more than 90% of these patients were willing to have their follow-up appointments through the same method. Moreover, all patients in the telemedicine group agreed that telemedicine saved money compared to in-person visits.

Over the past year, many epilepsy centers worldwide have shared their experience in regard to patients' or families' perception of telemedicine usefulness either in adults and pediatrics. High levels of satisfaction over 85% have been consistently noted in all studies (6, 8, 14, 15). Given the elevated satisfaction level noted among healthcare providers as well (9, 16), we believe that even after the pandemic, most epilepsy centers will implement telemedicine as part of their routine care. However, some limitations related to telemedicine should be recognized, including unexpected technical issues, lack of an appropriate reimbursement policy, impossibility to perform a full neurological exam, and lack of privacy for teenagers when attending visits from their parents' house (17).

Finally, when it comes to what families consider relevant in telemedicine, families from both centers agreed upon data safety, easy access to services, and consistency of services offered by the same familiar healthcare provider. A shorter waiting time was most relevant in Calgary, where longer waiting times exist. Likewise, timely appointments were nearly 100% rated as important or very important in Calgary for families to consider telehealth beyond the pandemic. Another relevant aspect consistently graded as important in both centers was their access to specialized care, while economic aspects were not seen as important for most families. Given the complexity of patients seen in both centers, these data suggest that families would prioritize telemedicine in order to keep their children's follow-up linked to epilepsy specialists and specialized centers.

Our study has some limitations. The patient samples from both centers may not represent newly diagnosed children with epilepsy, given that more than 80% of our families reported seizures for more than 12 months. Therefore, it is uncertain whether the level of parental anxiety in newly diagnosed cases changed with telemedicine, as our sample size did not allow us to explore this further. Furthermore, patient symptom evaluation consisted of a pure description of parental perception rather than objective measures or scales. In order to confirm our findings, future studies should use standardized methods to measure anxiety levels in parents and patients before and after telemedicine experience. Among several tools, the State–Trait Anxiety Inventory (STAI) has been translated and adapted in 48 languages (18). In addition, the Epilepsy Anxiety Survey Instrument (EASI) can be used specifically for people with epilepsy (19). One intrinsic issue from survey studies is the non-response bias. Our study might have a nearly 60% non-response bias if the opinions of non-responders differ substantially from those of responders. In addition, there is also a potential selection bias, given that the families who agreed to participate in an online survey are likely to have better knowledge and acceptance of telemedicine overall.

## Conclusions

Some children with epilepsy and their families have been negatively impacted by the pandemic, including worsening of overall health and behavior, and increased seizures. Independent of healthcare system and cultural surroundings, our data suggest that telemedicine can be helpful in managing epilepsy, and it might reduce parental anxiety levels. In our experience, families who used telemedicine were more positive toward similar future appointments. Despite potential barriers, telemedicine use in pediatric epilepsy is a valuable care alternative for patients and healthcare providers, and it is likely to continue post pandemic.

## Data Availability Statement

The original contributions presented in the study are included in the article/Supplementary Material, further inquiries can be directed to the corresponding author/s.

## Ethics Statement

The studies involving human participants were reviewed and approved by University of Freiburg Ethic Review Board, University of Calgary Ethic Review Board. Written informed consent to participate in this study was provided by the participants' legal guardian/next of kin.

## Author Contributions

All authors met the International Committee of Medical Journal Editors authorship criteria and had full access to relevant data. Neither honoraria nor payments were made for authorship. All authors contributed to the study concept and design and interpretation of the data. KK performed the statistical analysis. All authors contributed to manuscript revision and read and approved the submitted version.

## Conflict of Interest

The authors declare that the research was conducted in the absence of any commercial or financial relationships that could be construed as a potential conflict of interest.
