# Supplementary material for: Telehealth for Children With Epilepsy Is Effective and Reduces Anxiety Independent of Healthcare Setting
Source: Front Pediatr. 2021 Jun 10;9:642381. doi: 10.3389/fped.2021.642381 (PMC8222691; doi:10.3389/fped.2021.642381)
Supplement: Supplementary file 1 [file Data_Sheet_1.PDF]

Consent Form

UNIVERSITY OF CALGARY - CONSENT FORM TO PARTICIPATE IN RESEARCH

TITLE: Telehealth for families with epilepsy during the COVID pandemic
SPONSOR: The Alberta Children's Hospital Research Institute (ACHRI)
INVESTIGATOR: Dr. Julia Jacobs-Levan

This consent form is only part of the process of informed consent. It should give you the basic idea of what the research is about, and what your participation will involve. If you would like more detail about something mentioned here, or information not included here, please ask. Take the time to read this carefully and to understand any accompanying information.

INTRODUCTION
Dr. Julia Jacobs-Levan and associates from the Department of Pediatrics at the University of Calgary are conducting a research study to improve the services provided by the Pediatric Epilepsy Program.
The COVID pandemic has forced all of us to change our daily routines and has especially affected the health care system. Part of these changes is that many of our outpatient clinics have been closed and we are currently not able to support families in the same way we used to. We understand that this can be difficult for families with children that experience seizures.

WHY IS THIS STUDY BEING DONE?
The purpose of this research study is to improve our services and better understand you or your child's medical needs. We designed a questionnaire about your experience and thoughts regarding Telehealth. Even if you don't have experience with a remote

consultations yet, you can participate in this survey. We are asking you to support our efforts to optimize our care in difficult times.

HOW MANY PEOPLE WILL TAKE PART IN THIS STUDY?
About 450 people will take part in this study through the University of Calgary.

WHAT WILL HAPPEN IF ME OR MY CHILD TAKES PART IN THIS RESEARCH STUDY?
We are asking you to complete a one-time survey, which should take approximately 10 minutes to complete. The survey results are anonymous, and we do not collect any identifying information from you.

ARE THERE ANY POTENTIAL RISKS OR DISCOMFORTS I CAN EXPECT FROM THIS STUDY?
There are no known risks to you or your child by participating in this study. All data collected from the information you provide in the surveys will be kept confidential and anonymous, and results will be presented in aggregate form only.

ARE THERE ANY POTENTIAL BENEFITS IF I PARTICIPATE?
If you agree to participate in this study there may or may not be a direct benefit to you or your child.

Although participants may not perceive any direct personal benefit during the study, the information you provide will be indispensable in helping us understand the issues affecting our patients, so that we can ultimately improving the quality of care delivered to patients.

WHAT OTHER CHOICES DO I HAVE IF I DO NOT PARTICIPATE?
Participating in this study is completely optional. Do not participate if you do not want to. Your decision will not affect the standard medical care you or your child receives at the Alberta Children's Hospital.

WITHDRAWAL OF STUDY DATA
We do not record any identifying information from you, and because of this, we cannot link survey results to a specific individual. Therefore, the researchers cannot remove your data from the study once you have completed the survey.

WILL I BE PAID FOR PARTICIPATING, OR DO I HAVE TO PAY FOR ANYTHING?
You will not receive any form of compensation for your participation in this research study. You do not have to pay anything to take part in the study.

WILL INFORMATION ABOUT MY PARTICIPATION BE KEPT CONFIDENTIAL?
Any information that is collected from survey responses will remain completely confidential. We are not collecting any information that could be used to identify you or your child, and only summarized data will be used in any publication connected to this research.

Please note that authorized representatives from the University of Calgary and the Conjoint Health Research Ethics Board may look at the full dataset held at the Alberta Children's Hospital for quality assurance purposes.

HOW LONG WILL INFORMATION FROM THE STUDY BE KEPT?
Data collected in this study will be kept for a minimum of 5 years. Data collected for this study may be shared with other researchers for future studies that are unknown at this time. Any data shared with other researchers, will not include your name or other personal identifying information.
Any future use of this research data is required to undergo review by a Research Ethics Board.

HOW CAN I FIND OUT ABOUT THE STUDY RESULTS?
Results of the study can be made available to any participant that would like them. Please contact the research team at (403)955-2296 for this information.

SIGNATURES
By selecting 'Yes, I will be in this research study' on this page, you are acknowledging that you have understood to your satisfaction the information regarding your participation in the research study and agree to participate as a participant. In no way does this waive your legal rights nor release the investigators, or involved institutions from their legal and professional responsibilities. If you have further questions concerning matters related to this research, please contact:

Dr. Julia Jacobs-Levan 403-955-2296; email: julia.jacobs-levan@albertahealthservices.ca

If you have any questions concerning your rights as a possible participant in this research, please contact the Chair of the Conjoint Health Research Ethics Board, University of Calgary at 403-220-7990.

The University of Calgary Conjoint Health Research Ethics Board has approved this research study (REB20-0670).

Would you like to take part in this study?

- ○ Yes, I will be in this research study
- ○ No, I don't want to do this

Inclusion criteria

Was your child diagnosed with seizures or epilepsy?

- ○ Yes
- ○ No

Part 1 Demographics

What is your country of residence?

- ○ Canada
- ○ USA
- ○ United Kingdom
- ○ ________ Other (please specify)

How old is your child?

Please enter years and months

Years	
Months	

When did your child have the first seizure?

- Within the last month
- Within the last year
- 2-5 years ago
- More than 5 years ago

Does your child receive anti-epileptic medication for these seizures

- Yes
- No

Did you change treatment within the last 6 months?

- Yes
- No

Did your child experience seizures within the last 12 months?

- Yes
- No

How frequent are these seizures approximately?

- 1-6 per year
- 7-12 per year
- at least 1 per month
- at least 1 per week
- at least 1 per day
- more than 1 per day

Did your child ever experience tonic-clonic generalized seizures (big seizures)?

- No, never had
- Yes, but only in the past
- Yes, still has

Did your child ever experience a seizure lasting longer than 10 min?

- Yes
- No

Who is usually treats your child's epilepsy?

- mainly family physician
- mainly pediatrician
- mainly pediatric neurologist in the community
- mainly pediatric neurologist in hospital
- ______ Other (please specify)

How often do you usually see your neurologists for epilepsy care?

- At least once per month
- 3-4 times per year
- twice per year
- once per year
- Other (please specify)

Part 2: Your experience

In this part we will ask you questions relating to the changes in care that you experienced during the current COVID pandemic

Did you have a scheduled appointment or were waiting for one at the time when the pandemic started? If yes, was this appointment changed?

- No, I had no appointment planned
- Yes I had one planned and it was completely cancelled without alternative
- Yes I had one planned and it was cancelled with a new later appointment
- Yes I had one planned and I was offered a virtual, phone appointment
- Yes I had one planned and it took place as scheduled

Do you feel that the change in appointments has had an impact on your family?

- No, not acute effects.
- Yes, because a diagnosis/treatment was delayed
- Yes, because my questions could not be answered
- Other (please specify)

Did you have diagnostics scheduled at the time when the pandemic started? If yes, what happened?

- ○ No, I had no diagnostics scheduled
- ○ Yes, I had diagnostics planned but it was cancelled without alternative
- ○ Yes, I had diagnostics planned but we recieved a new, later appointment
- ○ Yes, I had diagnostics planned and it took place as scheduled

What kind of diagnostics has your child been scheduled for? (Please select all that apply)

- ☐ MRI
- ☐ EEG
- ☐ Longterm EEG
- ☐ PET
- ☐ SPECT
- ☐ ________ Other

Do you feel that canceled diagnostics has a negative impact on your child's health?

- ○ No, diagnostics can be done later
- ○ Yes we feel that the delayed diagnostics could seriously harm our child
- ○ Yes, because diagnostics are necessary to change/offer treatment
- ○ Yes, because we have to wait longer before getting a diagnosis
- ○ Yes, because we expect that the diagnostic results will clarify some of our questions

The current COVID pandemic has resulted in many restrictive measures. How do you feel your child's health has changed?

	Better	No change	Worse	Not applicable
Overall health	○	○	○	○

	Better	No change	Worse	Not applicable
Seizure frequency	○	○	○	○
Behavior	○	○	○	○

Part 3

In this part we would like to understand how we can optimally use virtual health to improve care during the pandemic.

Please indicate which technical equipment is available in your household (select all that apply)

- ☐ PC with camera
- ☐ Tablet with camera
- ☐ Wifi
- ☐ Phone
- ☐ Chat programs such as zoom, google hangout, skype

Did you use virtual/phone consultation with a physician or nurse for your child during the pandemic?

- ○ Yes
- ○ No

Who did you have the consultation with? (please select all options that apply)

- ☐ Registered Nurse (RN)
- ☐ Family physician
- ☐ Pediatrician

- [ ] Neurologist/pediatric neurologist
- [ ] ______ Other (please specify)

What medium did you use? (please select all options that apply)

- [ ] Phone
- [ ] Online with video (zoom, skype)
- [ ] Telehealth platform
- [ ] Email
- [ ] ______ Other (please specify)

Did you feel this consultation was helpful in times where in person consultations are not possible?

- ○ Yes
- ○ No

Please provide reasons or examples

______

In contrast to in patient appointments your virtual consultation was (select an option)

- ○ As efficient as an in-patient visit
- ○ Nearly as efficient as an in-patient visit
- ○ Partially helpful
- ○ Not at all helpful

Would you say that you fear that your child's epilepsy/seizures will worsen during the pandemic?

- Not at all
- A little bit
- Very much

Do you feel the appointment helped you to be less anxious?

- Not at all
- A little bit
- Very much

Did you have to come to the hospital despite having had a phone consultation?

- No
- Yes, because of an acute deterioration of seizures
- Yes, because of seizure-unrelated medical reasons
- Yes, because this was the recommendation after the virtual consultation

Please indicate how important the following aspects of health consultation are to you

	Very important	Moderately important	Not so important	Not at all important
Protection of my personal data	O	O	O	O
Easy access to program/medium	O	O	O	O

	Very important	Moderately important	Not so important	Not at all important
Ability to communicate face to face (via video)	○	○	○	○
Appointment without long wait time	○	○	○	○
Always with the doctor that knows my child	○	○	○	○

If you could chose a platform for future virtual consultations, which one would you use?

- ○ Phone
- ○ Online with video (zoom, skype)
- ○ Telehealth platform
- ○ Telehealth platform in a hospital setting
- ○ Email
- ○ ____________ Other (please specify)

Would you consider changing all or most of your appointments to this form if possible, even after the pandemic?

- ○ Yes, all
- ○ Yes, some
- ○ No, I prefer in person consultations

If the pandemic continues, how long do you feel you could handle your child's disease with virtual appointments only before having to come to the ED?

- ○ Less than 1 month
- ○ 2-6 months
- ○ 6-12 months
- ○ more than 1 year

Please indicate which would be reasons for you to consider virtual health beyond the time of the pandemic

	very important	moderately important	not so important	not at all important
Avoiding a long drive	○	○	○	○
Saving costs	○	○	○	○
Getting a timely appointment	○	○	○	○
Accessing specialized care	○	○	○	○
Seeing a specific physician	○	○	○	○

Do you wish to give any additional comments?

- ○ No, end survey
- ○ [ ] Yes, please comment

If you needed help with your child's epilepsy in the next time, who would you prefer having a virtual consultation with?

- ○ RN
- ○ Family physician
- ○ Pediatrician
- ○ Neurologist
- ○ [ ] Other (please specify)

Which medium would you prefer for this visit?

- Phone
- Online with video (zoom, skype)
- Telehealth platform
- Telehealth platform in a hospital setting
- Email
- [ ] Other (please specify)

Do you feel that this consultation could be helpful during the times of the pandemic?

- Yes
- No

Please give reasons

Do you think that in contrast to in patient appointments, your virtual consultation would be (select an option)

- As efficient as an in-patient visit
- Nearly as efficient as an in-patient visit
- Partially helpful
- Not at all helpful

Would you say that you fear that your child's epilepsy/seizures will worsen during the pandemic?

- Not at all

- A little bit
- Very much

Do you feel the appointment helped you to be less anxious?

- Not at all
- A little bit
- Very much

Please indicate how important the following aspects of a health consultation are to you

	Very important	Moderately important	Not so important	Not at all important
Protection of my personal data	O	O	O	O
Easy access to program/medium	O	O	O	O
Ability to communicate face to face (via video)	O	O	O	O
Appointment without long wait time	O	O	O	O
Always with the doctor that knows my child	O	O	O	O

Would you consider changing all or most of your appointments to this form if possible, even after the pandemic?

- Yes, all
- Yes, some
- No, I prefer in person consultations

If the pandemic continues, how long do you feel you could handle your child's disease with virtual appointments only before having to come to the ED?

- Less than 1 month
- 2-6 months
- 6-12 months
- more than 1 year

Please indicate which would be reasons for you to consider virtual health beyond the time of the pandemic

	very important	moderately important	not so important	not at all important
Avoiding a long drive	○	○	○	○
Saving costs	○	○	○	○
Getting a timely appointment	○	○	○	○
Accessing specialized care	○	○	○	○
Seeing a specific physician	○	○	○	○

Do you wish to give any additional comments?

- No, end survey
- [ ] Yes, please comment

Powered by Qualtrics
