# Supplementary material for: Telehealth for Children With Epilepsy Is Effective and Reduces Anxiety Independent of Healthcare Setting
Source: Front Pediatr. 2021 Jun 10;9:642381. doi: 10.3389/fped.2021.642381 (PMC8222691; doi:10.3389/fped.2021.642381)
Supplement: Supplementary file 2 [file Data_Sheet_2.PDF]

Telemedizin für Familien mit epilepsiekranken Kindern
- Erfahrungen aus der COVID-Pandemie

Liebe Eltern,

vielen Dank dass Sie bereits sind an unserer Umfrage zur Telemedizin teilzunehmen! Telemedizin umfasst Telefontermine genauso wie Videokonferenzen und ist in Zeit der Corona Pandemie sehr wichtig geworden. Wir würden gerne von Ihren erfahren, ob Sie auch Einschränkungen in der medizinischen Versorgung Ihres Kindes erlebt haben. Ziel ist es mit den Informationen der Umfrage unser Angebot an Telemedizin zu verbessern.

Die Teilnahme ist selbstverständlich freiwillig. Fragen können bis auf wenige Ausnahmen auch ausgelassen werden. Datenspeicherung und -auswertung erfolgen anonymisiert. Ein Rückschluss auf die Identität des Teilnehmers ist anhand der Daten nicht möglich. Nach Abschluss der Umfrage streben wir eine Veröffentlichung der gewonnen Erkenntnisse in einer medizinischen Fachzeitschrift an. Die veröffentlichten Daten sind nicht mit Teilnehmern verknüpft.

Für Rückfragen stehen wir sehr gerne zur Verfügung und danken Ihnen sehr herzlich für Ihre Teilnahme.

Mit freundlichen Grüßen,
Dr. A. Klotz
Leitung Kinderepilepsiezentrum Freiburg

Telemedizin für Familien mit epilepsiekranken Kindern
- Erfahrungen aus der COVID-Pandemie

* 1. Hat ihr Kind eine Epilepsie oder epileptische Anfälle?

- ◯ Ja
- ◯ Nein

Telemedizin für Familien mit epilepsiekranken Kindern
- Erfahrungen aus der COVID-Pandemie

Teil 1 Allgemeine Angaben

2. In welchem Land leben Sie?

- ◯ Deutschland
- ◯ Österreich
- ◯ Schweiz
- ◯ Luxemburg
- ◯ Frankreich
- ◯ Anderes (bitte angeben welches)

3. Wie alt ist Ihr Kind?

Jahre

Monate

4. Wann hatte Ihr Kind den allerersten Anfall?

- ◯ Innerhalb des letzten Monats
- ◯ Innerhalb des letzten Jahres
- ◯ vor 2-5 Jahren
- ◯ vor mehr als 5 Jahren

5. Bekommt Ihr Kind Medikamente gegen die Epilepsie?

- ◯ Ja
- ◯ Nein

6. Wurde die Therapie der Epilepsie in den letzten 6 Monaten umgestellt?

- ◯ Ja
- ◯ Nein

7. Hatte Ihr Kind in den letzten 12 Monaten Anfälle?

- ◯ Ja
- ◯ Nein

Telemedizin für Familien mit epilepsiekranken Kindern
- Erfahrungen aus der COVID-Pandemie

8. Wie häufig treten die Anfälle ungefähr auf?

- ◯ 1 bis 6 mal pro Jahr
- ◯ 7 bis 12 mal pro Jahr
- ◯ mindestens einmal im Monat
- ◯ mindestens einmal pro Woche
- ◯ mindestens einmal pro Tag
- ◯ mehrfach am Tag

9. Sind bei Ihrem Kind jemals große Anfälle ("Grand mal") aufgetreten?

- ◯ Nein, nie
- ◯ Ja, früher aber jetzt nicht mehr
- ◯ Ja, auch jetzt treten noch große Anfälle auf

10. Gab es bei Ihrem Kind jemals Anfälle, die länger als 10 Minuten gedauert haben?

- ◯ Ja
- ◯ Nein

11. Wer behandelt die Epilepsie Ihres Kindes normalerweise?

- ◯ unser Hausarzt
- ◯ unser Kinderarzt
- ◯ niedergelassener Neuropädiater oder Neurologe (in einer Praxis)
- ◯ Neuropädiater oder Neurologe in der Klinik, einem SPZ oder einem Epilepsiezentrum
- ◯ Sonstiges (bitte angeben)

12. Wie oft gehen Sie mit Ihrem Kind normalerweise wegen der Epilepsie zum Arzt?

- ◯ Mindestens einmal pro Monat
- ◯ 3-4 Mal pro Jahr
- ◯ Zweimal pro Jahr
- ◯ Einmal im Jahr
- ◯ Sonstiges (bitte angeben)

Telemedizin für Familien mit epilepsiekranken Kindern
- Erfahrungen aus der COVID-Pandemie

Teil 2: Ihre Erfahrungen

In diesem Teil möchten wir gerne mehr darüber erfahren ob und wie sich die medizinische Versorgung bezüglich der Epilepsie Ihres Kindes in der aktuellen Corona-Krise verändert hat.

13. Hatten Sie einen bereits geplanten Termin zur Kontrolle oder Behandlung der Epilepsie als die Pandemie begann? Falls ja, wurde der Termin geändert?

- ◯ Nein, es war kein Vorstellungstermin geplant
- ◯ Ja, ein Vorstellungstermin war bereits geplant und wurde ohne Alternative abgesagt
- ◯ Ja, ein Vorstellungstermin war bereits geplant, dieser wurde abgesagt und wir haben einen späteren Ersatztermin bekommen
- ◯ Ja, ein Vorstellungstermin war bereits geplant und mir wurde stattdessen ein Telefontermin angeboten.
- ◯ Ja, ein Vorstellungstermin war bereits geplant und konnte auch wie geplant stattfinden

Telemedizin für Familien mit epilepsiekranken Kindern
- Erfahrungen aus der COVID-Pandemie

14. Hatte die Änderung/der Wegfall des Termins einen negativen Einfluss auf Sie oder Ihr Kind?

- Nein, keine akuten Auswirkungen
- Ja, weil Therapie oder Diagnostik verspätet stattfinden
- Ja, weil ich meine Fragen nicht stellen konnte bzw beantwortet bekam
- Sonstiges (bitte angeben)

Telemedizin für Familien mit epilepsiekranken Kindern
- Erfahrungen aus der COVID-Pandemie

15. Waren diagnostische Untersuchungen geplant als die Pandemie begann? Falls ja, wurden diese durchgeführt?

- ◯ Nein wir hatte keinen Termin zur Diagnostik
- ◯ Ja, wir hatten schon einen Termin zur Diagnostik, dieser wurde aber ohne Alternative abgesagt
- ◯ Ja, wir hatten schon einen Termin zur Diagnostik, dieser wurde abgesagt und wir haben einen späteren Ersatztermin bekommen
- ◯ Ja, wir hatten schon einen Termin zur Diagnostik, dieser konnte wie geplant stattfinden

Telemedizin für Familien mit epilepsiekranken Kindern
- Erfahrungen aus der COVID-Pandemie

16. Für welche Art von Diagnostik waren Termine vereinbart? (bitte alles Zutreffende ankreuzen)

- [ ] Kernspintomographie=MRT
- [ ] EEG
- [ ] Langzeit EEG
- [ ] PET
- [ ] SPECT
- [ ] Sonstiges (bitte angeben)

17. Hat der abgesagte Termin zur Diagnostik nach Ihrer Einschätzung einen negativen Einfluss auf die Gesundheit Ihres Kindes?

- ◯ Nein, die Untersuchungen können später durchgeführt werden
- ◯ Ja, wir befürchten, dass sich durch die verzögerte Diagnostik der Gesundheitszustand unseres Kindes ernsthaft verschlechtern könnte
- ◯ Ja, weil die Untersuchungen benötigt werden um eine Behandlung zu beginnen oder die Therapie zu ändern
- ◯ Ja, weil wir länger auf eine Diagnosestellung warten müssen
- ◯ Ja, weil wir uns erhofften dass die Untersuchungsergebnisse weiterhelfen unsere Fragen zur Epilepsie unseres Kindes zu klären

Telemedizin für Familien mit epilepsiekranken Kindern
- Erfahrungen aus der COVID-Pandemie

18. Wie hat sich die Situation Ihres Kindes Ihrer Meinung nach geändert seit die Corona-Regeln gelten und die Kita/Schule geschlossen ist?

	Besser	Keine Veränderung	Schlechter	Für unser Kind nicht zutreffend
Gesundheitszustand insgesamt	○	○	○	○
Anfallsfrequenz	○	○	○	○
Verhalten	○	○	○	○

Telemedizin für Familien mit epilepsiekranken Kindern
- Erfahrungen aus der COVID-Pandemie

Teil 3

In diesem Teil würden wir gerne mehr darüber erfahren, wie wir Telemedizin optimalerweise einsetzen können um die Epilepsieversorgung während der Pandemie zu verbessern.

19. Welches technische Equipment ist in Ihrem Haushalt vorhanden (bitte alle Zutreffenden auswählen)

- ☐ Computer (PC oder Laptop) mit Kamera
- ☐ Tablet oder Smartphone mit Kamera
- ☐ Internetanschluss
- ☐ Telefon
- ☐ Chat Programme wie Zoom, Google hangout oder skype

* 20. Hatten Sie bereits einen Telefontermin oder Videotermin zur Epilepsie Ihres Kindes seit die Pandemie begann?

- ○ Ja
- ○ Nein

Telemedizin für Familien mit epilepsiekranken Kindern
- Erfahrungen aus der COVID-Pandemie

21. Mit wem haben Sie den Telefontermin geführt? (Mehrfachnennung möglich)

- [ ] spezielle Epilepsie-Krankenschwester
- [ ] Hausarzt
- [ ] Kinderarzt
- [ ] Neuropädiater oder Neurologe
- [ ] Sonstiges (bitte angeben)

22. Über welches Medium wurde der Termin durchgeführt? (bitte alle Zutreffenden auswählen)

- [ ] Telefon
- [ ] Online mit Video (Zoom, Skype oder andere)
- [ ] Spezielle Telemedizin Platform
- [ ] E-mail
- [ ] Sonstiges (bitte angeben)

23. Fanden Sie den Telefontermin hilfreich unter dem Aspekt dass persönliche Arztkontakte nur eingeschränkt möglich sind?

- ◯ Ja
- ◯ Nein

Was waren die Gründe?

24. Im Vergleich zu einem "echten" Arzttermin war der Telefontermin

- ◯ Genauso nützlich
- ◯ Fast genauso nützlich
- ◯ Nur teilweise hilfreich
- ◯ Nicht hilfreich

Telemedizin für Familien mit epilepsiekranken Kindern
- Erfahrungen aus der COVID-Pandemie

25. Befürchten Sie dass die Epilepsie bzw. die Anfälle Ihres Kindes sich während der Pandemie verschlechtern könnte(n)?

- ◯ Überhaupt nicht
- ◯ Etwas
- ◯ Sehr

26. Haben Sie das Gefühl durch den Telefontermin sind Sie weniger besorgt?

- ◯ Überhaupt nicht
- ◯ Etwas
- ◯ Sehr

27. Mussten Sie mit Ihrem Kind trotz Telefontermin in die Klinik kommen?

- ◯ Nein
- ◯ Ja, wegen einer akuten Verschlechterung der Epilepsie
- ◯ Ja, aber aus einem anderen Grund der nichts mit der Epilepsie zu tun hatte
- ◯ Ja, das war die Empfehlung aus dem Telefontermin

Telemedizin für Familien mit epilepsiekranken Kindern
- Erfahrungen aus der COVID-Pandemie

28. Bitte geben Sie an, wie wichtig Ihnen die folgenden Aspekte eines virtuellen Arzttermins sind

	sehr wichtig	ziemlich wichtig	nicht so wichtig	gar nicht wichtig
Datenschutz	◯	◯	◯	◯
Benutzerfreundliches Programm/Medium	◯	◯	◯	◯
Kommunikation mit Video	◯	◯	◯	◯
Keine lange Wartezeit	◯	◯	◯	◯
Immer mit dem gleichen Arzt	◯	◯	◯	◯

29. Welche Platform wäre Ihnen für einen virtuellen Arzttermin am liebsten?

◯ Telefon

◯ Online mit Video (zoom, skype)

◯ Spezielle Telemedizin Platform

◯ Spezielle Telemedizin Platform des betreuenden Krankenhauses (kein Fremdanbieter)

◯ E-Mail

◯ Sonstiges (bitte angeben)

30. Könnten Sie sich vorstellen, sofern möglich, auch nach der Pandemie Arzttermine virtuell wahrzunehmen?

- ◯ Ja, alle
- ◯ Ja, zum Teil
- ◯ Nein, persönliche Arztkontakte sind mir lieber

Telemedizin für Familien mit epilepsiekranken Kindern
- Erfahrungen aus der COVID-Pandemie

31. Sollte die Pandemie länger andauern: Wie lange wäre nach Ihrer Einschätzung eine rein virtuelle medizinische Betreuung der Epilepsie Ihres Kindes möglich (bevor Sie sich z. B. notfallmäßig vorstellen müssten)

- ◯ Weniger als 1 Monat
- ◯ 2-6 Monate
- ◯ 6-12 Monate
- ◯ über 1 Jahr

32. Bitte geben Sie an, welche der folgenden Gründe für Sie relevant wären damit auch nach der Pandemie virtuelle Arztkontakte in Frage kämen.

	sehr wichtig	ziemlich wichtig	nicht so wichtig	gar nicht wichtig
Vermeidung langer Fahrtstrecken	◯	◯	◯	◯
Kostenersparnis	◯	◯	◯	◯
Telefontermin früher zu bekommen als normalen Ambulanztermin	◯	◯	◯	◯
Zugang zu speziellen Epilepsieabteilungen zu haben (die ich sonst aufgrund z. B. Entfernung nicht erreichen könnte)	◯	◯	◯	◯
Einen speziellen Arzt zu sprechen	◯	◯	◯	◯

33. Haben Sie noch weitere Anmerkungen für uns?

- ◯ Nein, ich möchte den survey beenden
- ◯ Ja, folgende.....

Anmerkungen

Telemedizin für Familien mit epilepsiekranken Kindern
- Erfahrungen aus der COVID-Pandemie

34. Wenn Sie in nächster Zeit einen virtuellen Termin wegen der Epilepsie Ihre Kindes bräuchten, mit wem würden Sie diesen am liebsten vereinbaren?

- ◯ Spezialisierte Epilepsiekrankenschwester
- ◯ Hausarzt
- ◯ Kinderarzt
- ◯ Kinderneurologe oder Neurologe
- ◯ Sonstiges (bitte angeben)

35. Welche Platform wäre Ihnen für einen virtuellen Arztkontakt am liebsten?

- ◯ Telefon
- ◯ Online mit Video (zoom, skype)
- ◯ Spezielle Telemedizin Platform
- ◯ Spezielle Telemedizin Platform in einem Krankenhaus (kein Fremdanbieter)
- ◯ E-Mail
- ◯ Sonstige (bitte angeben)

36. Glauben Sie, dass ein virtueller Arztkontakt während der Pandemie hilfreich für Sie sein könnte für den Umgang mit der Epilepsie Ihres Kindes?

- ◯ Ja
- ◯ Nein

Bitte nennen Sie Gründe für Ihre Antwort

37. Wie schätzen Sie den Nutzen eines virtuellen Arztkontaktes im Vergleich zu einem "echten Arzttermin" ein?

- ◯ Genauso nützlich
- ◯ Fast genauso nützlich
- ◯ Teilweise hilfreich
- ◯ Überhaupt nicht hilfreich

Telemedizin für Familien mit epilepsiekranken Kindern
- Erfahrungen aus der COVID-Pandemie

38. Befürchten Sie dass die Epilepsie bzw. die Anfälle Ihres Kindes sich während der Pandemie verschlechtern könnte(n)?

- ◯ Überhaupt nicht
- ◯ Etwas
- ◯ Sehr

39. Haben Sie das Gefühl dass ein virtueller Arzttermin die Sorge reduzieren würde?

- ◯ Überhaupt nicht
- ◯ Etwas
- ◯ Sehr

40. Bitte geben Sie an, wie wichtig Ihnen die folgenden Aspekte eines virtuellen Arzttermins sind

	Sehr wichtig	Ziemlich wichtig	Nicht so wichtig	Gar nicht wichtig
Datenschutz	◯	◯	◯	◯
Benutzerfreundliches Programm/Medium	◯	◯	◯	◯
Kommunikation mit Video	◯	◯	◯	◯
Früheren Termin zu erhalten	◯	◯	◯	◯
Immer mit dem gleichen Arzt	◯	◯	◯	◯

Telemedizin für Familien mit epilepsiekranken Kindern
- Erfahrungen aus der COVID-Pandemie

41. Könnten Sie sich vorstellen, sofern möglich, auch nach der Pandemie Arzttermine virtuell wahrzunehmen?

- Ja, alle
- Ja, manche
- Nein, ich bevorzuge persönliche Arztkontakte

42. Sollte die Pandemie länger andauern: wie lange wäre nach Ihrer Einschätzung eine rein virtuelle medizinische Betreuung der Epilepsie Ihres Kindes möglich (bevor Sie sich z. B. notfallmäßig vorstellen müssten)?

- Weniger als 1 Monat
- 2-6 Monate
- 6-12 Monate
- über 1 Jahr

43. Bitte geben Sie an, welche der folgenden Gründe für Sie relevant wären damit auch nach der Pandemie virtuelle Arztkontakte in Frage kämen.

	Sehr wichtig	Ziemlich wichtig	Nicht so wichtig	Gar nicht wichtig
Vermeidung langer Fahrtstrecken	◯	◯	◯	◯
Kostenersparnis	◯	◯	◯	◯
Telefontermin früher zu bekommen als normalen Ambulanztermin	◯	◯	◯	◯
Zugang zu speziellen Epilepsieabteilungen zu haben (die ich sonst aufgrund z. B. Entfernung nicht erreichen könnte)	◯	◯	◯	◯
Einen speziellen Arzt zu sprechen	◯	◯	◯	◯

44. Haben Sie noch weitere Anmerkungen für uns?

◯ Nein, ich möchste den Survey beenden

◯ Ja, folgende....

Anmerkungen

Telemedizin für Familien mit epilepsiekranken Kindern
- Erfahrungen aus der COVID-Pandemie

Vielen herzlichen Dank für Ihre Teilnahme!
